# Rheological and Textural Investigation to Design Film for Packaging from Potato Peel Waste

**DOI:** 10.3390/gels10110681

**Published:** 2024-10-23

**Authors:** Olga Mileti, Noemi Baldino, Vittoria Marchio, Francesca R. Lupi, Domenico Gabriele

**Affiliations:** 1 Department of Information, Modeling, Electronics and System Engineering (D.I.M.E.S.), University of Calabria, Via P. Bucci, Cubo 39C, I-87036 Rende, Italy; o.mileti@dimes.unical.it (O.M.); francesca.lupi@unical.it (F.R.L.); d.gabriele@unical.it (D.G.); 2 Department of Pharmacy, Health and Nutritional Sciences, University of Calabria, Via Pietro Bucci, I-87036 Rende, Italy; vittoriamarchio@gmail.com

**Keywords:** biopackaging, viscosity, agrifood, rheology, polyphenols

## Abstract

The recovery of potato waste for circular-economy purposes is a growing area of industrial research. This waste, rich in nutrients and potential for reuse, can be a valuable source of starch for packaging applications. Rheology plays a crucial role in characterizing film-forming solutions before casting. In this work, packaging film was prepared from potato waste using rheological information to formulate the film-forming solution. To this aim, rheological measurements were carried out on starch/glycerol-only samples, and the data obtained were used to optimize the formulation from the waste. The polyphenol content of the peels was analyzed, and the resulting films were comprehensively characterized. This included assessments of color, extensibility, Fourier-transform infrared (FT-IR) spectroscopy, surface microscopy, and contact angle. Polyphenol-loaded films, suitable for packaging applications, were developed from potato waste. These films exhibited distinct properties compared to those made with pure starch, including an improved wettability of about 75° for the best sample and a high elastic modulus of about 36 MPa, which reduces the deformability but enhances the resistance against the stress. Through rheological studies, we were able to design films from potato peel waste. These films demonstrated promising mechanical performance.

## 1. Introduction

The use of industrial waste, in particular from the agricultural and food industries, represents an ambitious goal for the circular economy [1]. The focus on the use of alternatives to plastics is increasing due to the strong environmental impact that plastics have [2]. Agri-food sources, including processing waste, are, in many cases, compounds rich in protein species, polysaccharides, and/or bioactive substances, which can be recovered and reused in various fields of application [3]. It is therefore important to aim at the recovery of these components from food waste by using them in the most suitable areas. In particular, potato peels are one of the most interesting waste products due to the different components contained within them, which can therefore be recovered and valorized [4,5]. They have an important nutritional value and are mainly composed of starch, fiber, and protein [6]. The peels contain nutritionally and pharmaceutically interesting components, such as phenolic compounds, which can be used as natural antioxidants capable of capturing free radicals and preventing oxidation reactions in edible oils [7]. In fact, potato skin is rich in polyphenols, with levels that are 10 times higher than those found in the flesh and account for 50 percent of all polyphenols contained in potatoes [8]. Polyphenols in potatoes are mainly phenolic acids, flavonoids, and anthocyanins [4,8]. For this reason, they can be thought of as natural antioxidants, capable of preventing the oxidation of lipids in food [9]. Potato peel waste, after fermentation, extraction, and/or other treatments, can also be used to obtain biofuels, dietary fibers, biofertilizers, biogas, antioxidants, and food additives [10]. In particular, the presence of starch in them allows for the activation of fermentation phenomena, but to be converted into sugar, saccharification (the hydrolysis of carbohydrates) is required so that the starch is available for the fermentation process [10]. Another possible use is the utilization of potato peels as a substrate to produce animal feed or biogas, although this type of reuse represents a devaluation and loss of the nutrients within them, which can instead be recovered and revalorized in more interesting areas [10]. There are studies that support the use of potato peels as a source of insoluble fibers tested as cholesterol-lowering agents or as a source of anti-carcinogenic substances [10,11,12]. Other studies suggest the use of potato peels with wheat flour for the production of biscuits and muffins [10,13]. Other studies support the use of starch extracted from potato for the preparation of food films [14,15,16,17,18], which can be improved through the use of ultrasound, having an incisive effect on the films obtained, leading to films with lower water vapor permeability and water solubility, and with higher mechanical strength and elongation capacity [19].

Given the composition of potato peels, traditionally considered waste, we decided to repurpose them and explore their potential value, drawing inspiration from existing research [20]. 

The research aims to develop a viable and environmentally friendly packaging material, and the aim is pursued through a rheological investigation, analyzing the thermal ramp viscosity measurements and the gelatinization points. 

Building upon previous research that identified an optimal starch/glycerol suspension for producing high-strength packaging films, we explored the potential of using potato peel waste as a sustainable alternative within a circular-economy framework. Our study focused on determining the optimal amount of starch to add to the formulation by using rheological tests to calculate the appropriate quantity.

## 2. Results and Discussion

### 2.1. Film-Forming Solution Rheology

**Figure 1:**
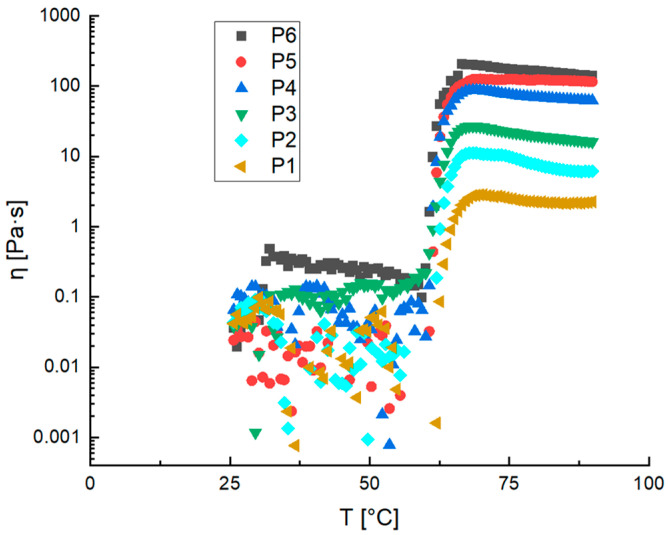
SSRTRT of starch-based film-forming solution. The values of viscosity (η) are reported as the temperature (T) changes for all samples of potato starch from pulp (P), with starch concentrations of 1% *w*/*w* (P1), 2% *w*/*w* (P2), 3% *w*/*w* (P3), 4% *w*/*w* (P4), 5% *w*/*w* (P5), 6% *w*/*w* (P6).

Starch/glycerol solutions, with different starch quantities (1–6% *w*/*w*), but at the same starch/glycerol ratio, were studied using Shear Step Rate temperature Ramp Test (SSRTRT) measurements, and the results are reported in Figure 1, in terms of viscosity (η), as the temperature (T) changes.

The trend in the curves in Figure 1 is the same for all formulations. An almost constant viscosity value was found for all the samples in the temperature range between about 25 and 60 °C. The viscosity value at low temperatures varies between 0.01 and 0.6 Pa s and increases as the amount of starch in the formulation increases. Around 60 °C, a clear increase in the viscosity was observed for all the samples studied, related to the gelatinization phenomena, leading to the formation of a highly viscous starch gel [16]. Gelatinization of potato starch leads to the disruption of hydrogen bonds and granule structure. The birefringence and crystalline order characteristic of the dispersion phase are lost, and a gel with markedly defined viscosity characteristics is formed [21].

As it is possible to observe from Figure 1, after gelatinization the viscosity increases reaching a maximum value after which remains almost constant. The velocity of viscosity increment is the gelatinization rate, which is related to the starch content in the samples. It is evident from the trend reported in Table 1 that the gelatinization rate increases increasing the starch content as well as the viscosity value at 90 °C, showing a clear correlation between the starch quantity in the film-forming solution (FFS) and the final gel viscosity. As the starch content increases, the rate of starch gelatinization accelerates (See Table 1). However, the gelatinization temperature remains relatively constant.

**Figure 2:**
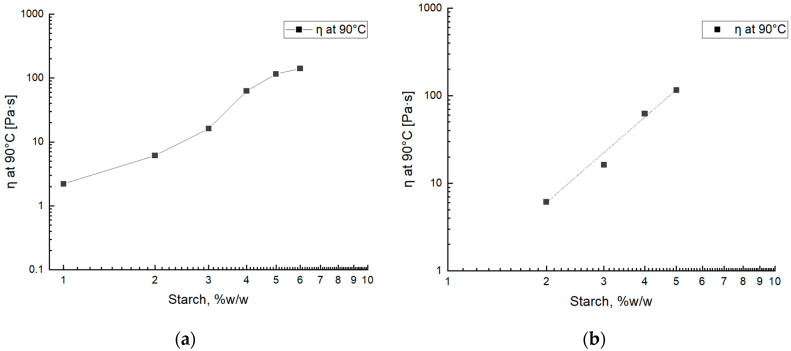
Viscosity (η) results of SSRTRT at 90 °C of the gelatinized samples (**a**) for all samples P1, P2, P3, P4, P5, and P6 and the calibration curve evaluated only in the linear zone with P2, P3, P4, and P5 samples (**b**).

The gel viscosity of the samples was reported in Figure 2a as a function of the starch concentration. The viscosity trend shows an almost sigmoidal trend with a linear zone in the concentration range between 2 and 5% *w*/*w*. Because the FFSs at low (1% *w*/*w*) and high starch concentrations (6% *w*/*w*) have given weak and too-strong films, respectively, it was decided to fit the zone between 2 and 5% *w*/*w* of starch concentration. The data and the films obtained are in agreement with the literature [15,17] because the viscosity of the film-forming solution is in the range of 0.001–100 Pa·s, which is similar to other starch-based samples investigated in previous works [15,17]. Moreover, the values of the gelatinization temperature obtained are similar to the values found in previous works [15,21].

As it is possible to observe in Figure 1, at low temperatures, the solution viscosity is low, and above the gelatinization temperature, the viscosity increases significantly, with a sharp increment. To correlate the viscosity of film-forming solutions prepared from waste peels with their starch content at 90 °C, a graph was plotted depicting the relationship between viscosity and starch concentration for all samples (Figure 2a).

It is possible to observe that the viscosity increases linearly only between 2 and 5% *w*/*w* of starch quantity. Then, because the better mechanical properties are for film-forming solutions within this range, a calibration curve was obtained only between 2 and 6% *w*/*w* of starch.

The trend was interpreted with a power law as reported in Figure 2b.

The power law obtained is the following:

(1) $$\eta =0.55x^{3.32}$$

This Equation was used as a calibration curve to evaluate the real starch concentration when the potato peel waste and wastewater were used to obtain FFSs. The evaluation was conducted by comparing the gel viscosity value at 90 °C with the calibration curve.

### 2.2. Rheology of Samples from Potato Waste

Following the procedure described in Section 2, FFSs were prepared using the powder obtained from the extraction of the potato peel waste. 

**Figure 3:**
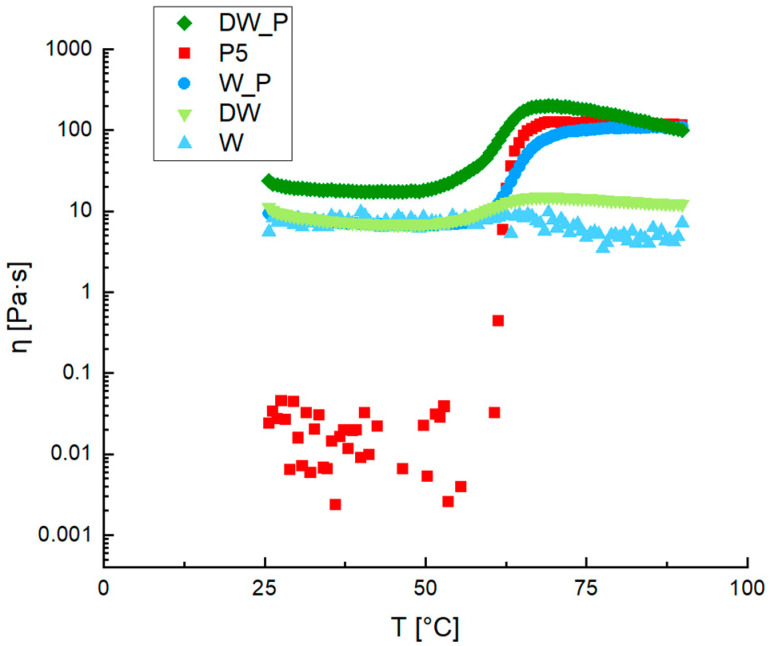
SSRTRT of film-forming solution, in terms of viscosity (η) as temperature (T) changes, obtained from potato wastes with (W_P and DW_P) and without (W and DW) starch addition, in comparison with the P5 sample.

Formulations obtained by extracting starches from blanched potato peels by adding distilled water (DW) and from blanched potato peels by using the same boiling water (W) were studied. The two different ways to prepare the films were investigated to valorize the waste of the agro-food industry. As reported in the literature, in fact, potato peels are rich in nutrients, which can be in part extracted in water during boiling [9]. The results of the rheological measurements are reported in terms of viscosity versus temperature in Figure 3. The P5 viscosity behavior is also reported because it represents the ideal formulation to obtain the film target already studied in previous works [15,16]. 

The W and DW suspensions are characterized by a higher viscosity than the P5 suspension at 25 °C and in the temperature range of 25–60 °C. This may be related to all species precipitated during the starch extraction process which tends to give the suspension consistency even before gelatinization. Unexpectedly, the viscosity of W and DW samples did not increase significantly following gelatinization, suggesting a low starch content in the extract derived from blanched skins [15,16,22].

Due to the low viscosity of samples W and DW following gelatinization, additional starch was incorporated to achieve the desired final viscosity. The required starch quantity was determined using Equation (1), resulting in samples W_P and DW_P. As illustrated in Figure 3, samples DW_P and W_P attained the final viscosity of the P5 sample. However, the sample prepared with distilled water consistently exhibited higher viscosity values than P5, while W_P demonstrated a viscosity trend closely resembling the reference sample after 60 °C.

### 2.3. Film Results

**Figure 4:**
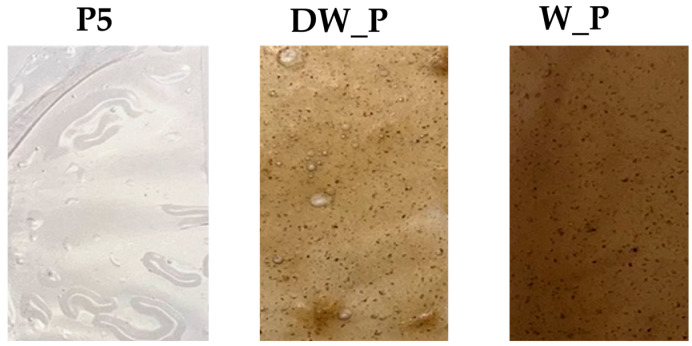
The visual aspect of the three prepared film samples obtained by camera.

Film samples were prepared with the optimized-waste film-forming solutions. The images of the films obtained are shown in Figure 4 for samples DW_P and W_P in comparison with sample P5.

Visual inspection revealed a brown color for the films derived from waste materials. This characteristic could be beneficial in applications where the film’s primary function is to shield the product from external light, as is often required for packaging and other products.

From the color measurements, a quantitative evaluation of all samples studied was performed, and the results are shown in Table 2.

L*, a*, and b* were evaluated with a colorimeter, and, in particular, L* is the brightness-axis variable from white to black, a* shows the variation from green to red, and b* from blue to yellow. The color variation of the samples is marked when the film is created with the addition of starch from waste. All the color parameters are different from those found for sample P5. In particular, a decrease can be observed in L* which visually translates into a more opaque appearance of the film; an increase in a*, indicative of an increase in reddish pigmentation, is clearly detectable in the skin used; an increase in b*, relating to the intensification of the yellow color typical of the potato, is observed. 

From the film thickness measurements, it can be seen that preparing films from waste leads to thicker films, particularly in the case of the W_P and DW_P samples, i.e., samples with starch added. 

The solubility is 12.13 ± 0.20% for the P5 sample and increases for the sample obtained with potato peel waste and glycerol addition. In particular, the sample DW_P has a value of 29.04 ± 0.30%, and the sample W_P has a value of 22.71 ± 0.15%. The potential food applications may require good water insolubility to enhance product integrity and water resistance, and the value found for the sample obtained with wastewater is lower compared to the value obtained for other films with potato waste.

The mechanical properties of tensile strength were evaluated by means of elongation measurements from which the values of percentage elongation at break (EAB%) and elastic modulus (E) were obtained, which are shown in Table 2. It could be observed that the reference sample, P5, has a high deformation capacity and low elastic modulus. Samples prepared without the addition of starch showed low deformability (low EAB% values) in both films prepared with (W) or without (DW) waste water. In both cases, the elastic modulus values are high, highlighting the stiffness of the films, which is probably related to the presence of other species recovered during starch extraction and which have a filler effect within the prepared film but do not have a plasticizing effect. The addition of starch makes it possible to obtain films that are able to elongate more and are less rigid, resulting in a better performance for an eventual printing process. The W_P sample was not characterized by elongational kinematics because it was particularly resistant to elongation. The range of values for EAB% and elastic modulus obtained from packaging film made from potato waste is generally comparable to those found in this study [23,24,25].

Contact angle measurements were carried out on all films prepared with potato waste, both on the surface in contact with the substrate and on the evaporation surface. Table 3 shows the results, from which it is observed that the evaporation surface is always more hydrophobic than the casting surface. The differences between the casting and evaporation surfaces might result from the humidity gradient that forms during the evaporation process. This issue could be resolved by refining the evaporation technique, such as rotating the film at a specific point. In every case, the contact angle increases for all the samples with potato peel waste compared to the P5 angle value. Furthermore, it is observed that the addition of starch to the formulations leads to more hydrophobic films, probably as a result of bonds established in the matrix. 

Comparing the results with what was obtained in the literature, it is observed that the contact angle values obtained are similar to the values reported by other authors for films obtained from potato peels, confirming a hydrophilic behavior in this type of film [23,26]. Even if the plasticizer’s presence is small, it interferes with the starch bond (hydrogen bond) and exposes hydroxyl groups of starch, lowering hydrophobicity [23,27].

### 2.4. Polyphenol Extraction and FT-IR Analysis

Using spectrophotometric analysis, the amount of polyphenols in the potato waste used was evaluated. The concentration of polyphenols present is 0.564 ± 0.02 mg GAE/g dw. The value obtained is in line with what is reported in the literature using different measurement techniques [28]. In particular, using fresh peels, the literature values are higher and around 3 mg GAE/g dw [6,29]. The presence of polyphenols in the extract is particularly interesting with a view to its potential use in films. Indeed, the presence of polyphenols could make the film antioxidant-active [30,31]. The inclusion of polyphenols in food films could make the packaging “active” and therefore increases the effectiveness of the packaging itself. Since polyphenols are naturally present in peel waste, this by-product of the food industry can be an important source of antioxidant materials to be used, acquiring economic value [4]. In order to verify the presence of polyphenols on the film prepared from potato peel waste, FT-IR analysis was carried out on the various samples studied, in particular, both on the polyphenol extract from the peel and on the prepared films, as well as on the potato peels as such and after blanching.

**Figure 5:**
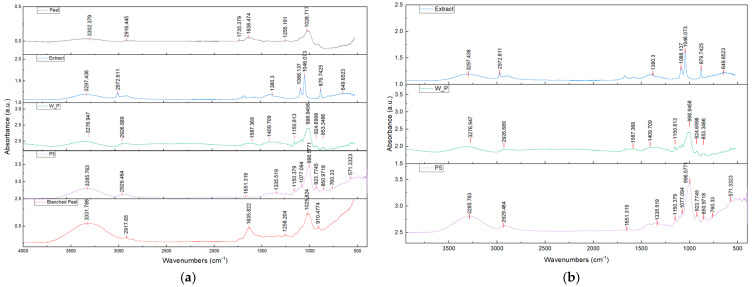
FT-IR results on the tested samples reported as Absorbance vs. Wavenumber. In particular, from the top of this Figure, (**a**) P5, W_P, extract from potato waste, blanched potato peel, and fresh potato peel. From the top of this Figure (**b**) P5, W_P, and extract from potato waste.

The FT-IR analysis revealed the presence of several characteristic peaks, which can be assimilated into the characteristic composition of the films. Spectra are shown in Figure 5, and the analysis was also carried out on the potato peel as is (PP) and after blanching (PP_T) to verify the matrix before and after heat treatment. As can be seen, the peaks are not lost as a result of the potato-blanching process, and, in detail, a peak is observed at 3331–3302 cm^−1^, relating to the presence of the hydroxyl groups of cellulose, lignin, and absorbed water. Also observed are the peaks at 1635 cm^−1^ and 1638 cm^−1^, ascribed to the vibration of the C=C and C=O groups, respectively [15,32,33], which may be related to the presence of flavonoids [34] in potato skins which resist the blanching process.

The spectrum analysis carried out on the film obtained with the W_P formulation shows that the characteristic peak at 1651 cm^−1^, which can be observed in the sample obtained from potato starch pulp according to the P5 formulation, is modified in the presence of the polyphenols by shifting the amplitude of this peak to a wider range of wavelengths.

In conclusion, the FT-IR analysis revealed the presence of polyphenols within the film produced from the extract obtained from potato peel, making it probably suitable for the development of active packaging.

### 2.5. SEM Images

**Figure 6:**
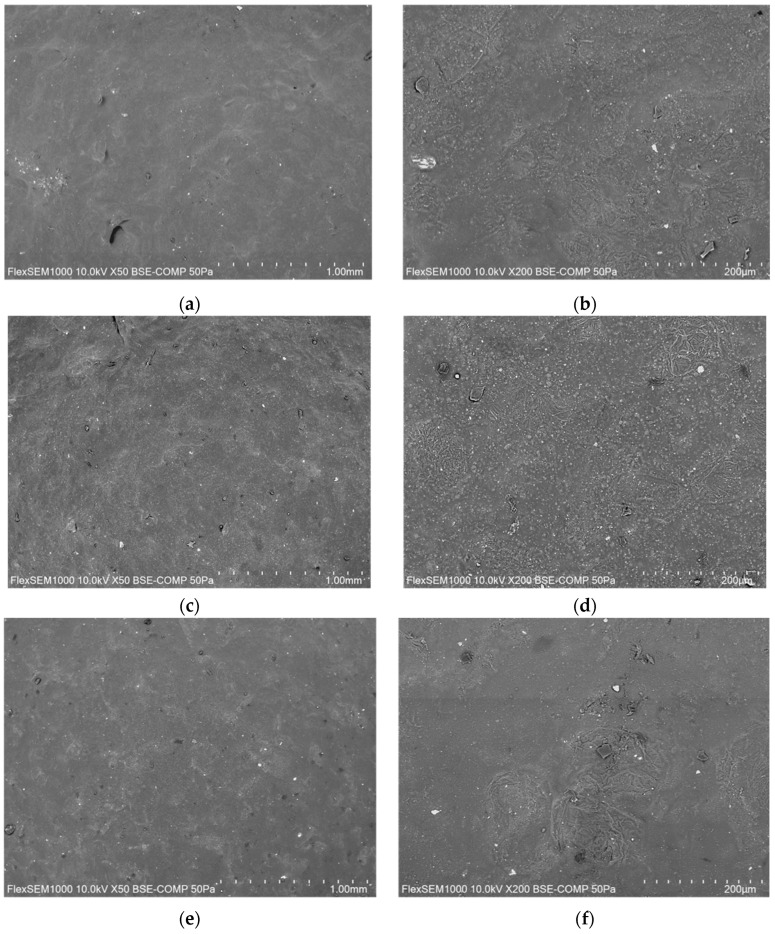

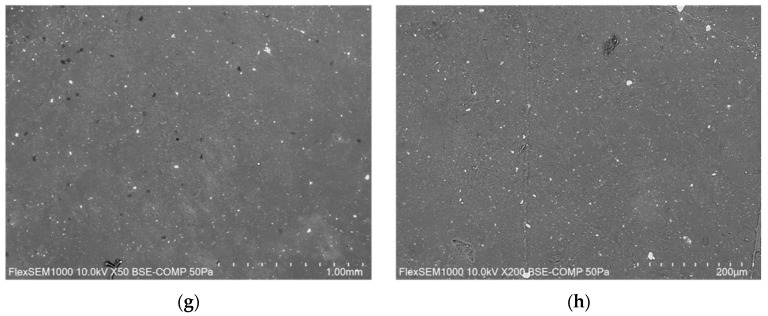
SEM images of W (**a**,**b**), WD (**c**,**d**), W_P (**e**,**f**), and WD_P (**g**,**h**) at 50× (**a**,**c**,**e**,**g**) and 200× (**b**,**d**,**f**,**b**).

The prepared films were subjected to microscopic analysis to investigate their surface properties. Below, Figure 6 shows images of the samples taken at 50× for an overview of the surface and a slightly more detailed one at 200×.

Analyses showed an irregular surface morphology, as expected from the use of a rather heterogeneous raw material such as potato peel extract. The solids extracted from potato peels contain, in fact, not only starch but also other species that make the surface of the film rough to the touch and also to observation. The use of potato peel extract makes surfaces rough, a quality that can also be felt by touch. This effect was also observed by other researchers when preparing edible films from potato peels [26]. Additionally, using distilled water in the preparations (Figure 6c,g) seems to make the surface more uneven, while using the same cooking water (Figure 6a,e) results in a more homogeneous surface.

## 3. Conclusions

This study developed a film for packaging using post-blanching potato peels as a sustainable material. The design of the film was performed by analyzing the rheological properties of standard films made from potato starch and glycerol. 

These peels are valuable due to their high content of functional substances like starch, cellulose, and polyphenols. To maximize resource utilization, we experimented with both blanched peels alone and peels combined with their boiling water to recover residual starch. 

Through the rheological study of the gels obtained to prepare the film-forming solutions, films with potato peel waste were formulated. 

The film’s design was made possible by analyzing viscosity curves at different temperatures and evaluating how post-gelatinization viscosity changes with varying starch content. This allowed us to obtain a calibration curve for the possible integration of starch in the film-forming solutions obtained with the waste.

The films obtained were found to be particularly resistant, and the characteristics were also investigated by means of FT-IR analysis, which confirmed the presence of polyphenolic compounds within the films, making them suitable for active packaging applications. The contact angle measurements also show an improved wettability compared to sample P5 for all four samples obtained with potato peel waste. The analysis also demonstrated that the blanching process did not compromise the antioxidant properties of the raw material. 

Our findings demonstrate that the sample prepared using distilled water, potato peel waste, and glycerol offers the most favorable combination of wettability, deformation, and strength. This suggests that this material warrants further research and development for potential applications.

## 4. Materials and Methods

### 4.1. Materials

Marabel’ potatoes from Sila (Calabria, Italy) were used for this study.

A 0.1 N Sodium hydroxide (AppliChem Panreac, Darmstadt, Germany) was used for starch precipitation. Distilled water, glycerol (J.T. Backer, Deventer, The Netherlands), and starch extracted from the various potato parts were used to formulate the filmogenic solutions and films.

Methanol (AppliChem Panreac, Darmstadt, Germany), Folin–Ciocalteu’s phenol reagent (Merck KGaA, Darmstadt, Germany), and gallic acid (Merck KGaA, Darmstadt, Germany) were used for polyphenol extraction and characterization.

### 4.2. Starch Extraction

Starch extraction was carried out following the procedure suggested in the literature [35] on blanched potato peels to simulate industrial waste and on the fresh potato without peel. In the first case, the potatoes were blanched in boiling water for 30 min; then, the skins were collected and stored in the freezer at −18 °C. Therefore, they were first defrosted for use. In total, 100 g of potato peels and 100 mL of water were mixed using a blender (Braun—MR 404 Plus, Esplugues de Llobregat, Spain) for 1 min and separated using a sieve to discard the larger pieces. The retentate was combined with an additional 100 mL of water, and the procedure was repeated two more times. The solution obtained was left to rest for 15 min. Then, to facilitate the precipitation of starches, 100 mL of 0.1 N sodium hydroxide (AppliChem Panreac, Darmstadt, Germany) was added, and the solution was left to rest for 1 h. The bottom was filtered to recover the starch. The mixture was dried in an oven (FD 53, Binder, Tuttlingen, Germany) at 40 °C for 7 h, and, at the end, the powder was collected. This procedure was applied to obtain the starch from potato peel waste, and the starch obtained was named W. The starch obtained from fresh potato without peel was obtained in the same way as W but without blanching and freezing. This last starch was named P. 

### 4.3. Film-Forming Solution and Film Preparation 

All the starch/glycerol formulations were prepared following the literature methodology [15,16]. A starch/glycerol ratio of 2/1 was used in all formulations.

The film-forming solution was prepared following a standard procedure: Starch, glycerol, and water were mixed using a magnetic stirring plate (AREX Heating Magnetic Stirrer, Velp Scientifica, Usmate Velate MB, Italy) at 25 °C for 5 min, and then, the mixture was sonicated using a sonicator (Bandelin Sonorex, RH 102 K, Berlin, Germany) for 10 min; subsequently, to improve starch dispersion, a homogenization step was carried out using a rotor–stator system (Ultraturrax, T50, IKA, Staufen, Germany) for 1 min at 2867× *g*. Finally, the system was heated up to 80 °C under mechanical stirring at 5× *g* (RW20, IKA, Germany) for 45 min using a thermal bath.

The suspension was used to obtain films using the tape-casting method at 40 ◦C by a knife (SAFA-209/3, SAMA Italia s.r.l, Viareggio, Italy). After casting, the film was dried for about 3 h at 75 °C in a forced-convection oven (FD 53, Binder, Tuttlingen, Germany). Once cooled, the film was gently removed from the surface.

Samples P1–P6 in Table 4 represent the samples prepared with potato starch (P) extracted from the pulp. The samples identified with the abbreviation W or W_P represent the samples prepared from waste and also including waste water. Finally, the samples prepared from waste (W), but with the addition of distilled water (D), are identified as DW and DW_P, while the samples prepared with waste but with the starch addition (P) are identified as W_P and DW_P. The starch addition in samples prepared from wastes was evaluated by the calibration law (Equation (1)) that correlates gel viscosity and starch concentration.

### 4.4. Extraction and Characterization of Polyphenols

The evaluation of polyphenols was carried out on the blanched potato peels according to the literature methods [29]. In particular, an indirect ultrasound extraction technique (IUAE) was used. The matrix was dried for 12 h at 50 °C in a ventilated oven (Binder FD 53, Germany) and then reduced to dust. Then, 10 g of powder was mixed with methanol (200 mL, 50% *v*/*v*) and placed in a sonicator (Bandelin Sonorex, RH 102 K, Germany) at 25 °C for 60 min at a vibration frequency of 43 kHz. After, the mixture was centrifuged at 4332× *g* for 30 min using a centrifuge (Centrifuge 5810 Eppendorf, Milano, Italy). The precipitate was resuspended in 100 mL of methanol (50%, *v*/*v*), and the procedure was repeated. The extracted amount was brought to dryness using a Rotavapor (Heidolph G3, Hei-VAP Value), and the extraction capacity was assessed via spectrophotometry using a UV-1601 spectrophotometer (Shimadzu, Duisburg, Germany), in terms of the amount of gallic acid equivalent per gram of loaded matrix, according to the Folin–Ciocalteu protocol [36].

### 4.5. Film Characterization

FFs were characterized by rheological measurements performed by a stress-controlled rheometer HAAKE MARS III (Thermo Scientific, Braunschweig, Germany) equipped with a parallel plate geometry (=50 mm, gap = 1.0 ± 0.1 mm). The suspensions, before the heating process, were characterized using Shear Step-Rate Temperature Ramp Rest (SSRTRT) measurements in a temperature range between 25 and 90 °C, using a heating ramp of 2 °C/min and a constant shear rate of 0.1 s^−1^, to evaluate the gelatinization temperature (Tgel, °C) [15,17]. Tgel was evaluated as reported in a previous work [15]. Furthermore, the gelatinization rate (Pa∙s/ °C) was evaluated as the slope of the linear region that characterizes the marked increase in viscosity in the curve of SSRTRT. During all the rheological tests, water loss was prevented by covering the sample rim with a low-viscosity silicon oil (viscosity 8.7 mPa·s, Sigma-Aldrich, Taufkirchen, Germany). 

All the investigated films were analyzed using a colorimeter (CR-400, Konica Minolta, Tokyo, Japan) to measure color in a CIE Lab color space. The parameters allowed for the objective evaluation of the color, which was defined using CieLab coordinates, where L* was the brightness axis, which varies from white to black, a* was the axis of the transition from green to red, and b* was the axis of the transition from blue to yellow. A white reference surface was used as a calibration surface. The tests were carried out on the evaporation surface. Colorimetric tests were performed in duplicate, and the results for each parameter are reported as mean value and standard deviation.

The surface morphology of the films was investigated using a scanning-electron microscope using FlexSEM 1000 II, HITACHI, Tokyo, Japan. The samples were placed on double-sided carbon tabs placed on an aluminum stub. All images were acquired under low-vacuum conditions (50 Pa), using a BSE-COMP back-scattered electron detector, with an acceleration voltage of 10 kV and a working distance of 8 mm. Morphological analysis was performed at 50× and 200× magnifications. 

Spectroscopy measurements were performed using a Nicolet iS-10 FT-IR spectrometer (Thermo Fisher Scientific, Carlsbad, CA, USA) with Smart iTX ATR module. Spectra were sampled in a wavelength range of 400–4000 cm^−1^ [16] in ATR. 

Elongational kinematics measurements were carried out to assess the mechanical properties of the prepared films, using a Zwick tensile machine (Zwick Roell Z005 TN, Ulm, Germany). The sample with dimensions of 100 mm × 20 mm was placed between the grips at a distance of 40 mm. The traverse rate used was 50 mm/min according to the literature [15,31], and the elongational stress recorded as the specimen deformed was measured. From the stress–strain curve, it was possible to assess the elasticity of the specimen (E) as the slope of the linear section of the curve and the maximum elongation at break (EAB%) value as the maximum elongation that the specimen can tolerate before failure [15,17].

Finally, contact angle measurements (θ) were made, using distilled water and studying the surface from both the evaporation and casting sides. Contact angle measurements were made using a pendant drop tensiometer (FTA200, First Ten Angstrom, Newark, CA, USA). 

### 4.6. Soluble Matter Determination

To determine the total soluble matter according to (Mileti 20224 tannini), we calculated the percentage of material released from the film packaging into distilled water. Before testing, the samples were preconditioned in an oven at 105 °C for 12 h. Then, 15 × 15 mm samples were weighed and immersed in 50 mL of distilled water. The water was stirred magnetically at 175 rpm and room temperature (22 ± 1 °C). After 12 and 24 h, the samples were removed, dried in an oven at 105 °C for 12 h, and reweighed. The soluble matter was calculated using Equation (2):

(2) $$SM\%=\frac{w_{i}-w_{f}}{w_{i}}\times 100$$

These solubility tests were conducted in triplicate, and the results are presented as average values with standard deviation.

## Figures and Tables

**Table 1:** Gelatinization temperature and rate for all samples.

ID Sample	T Gel, °C	Gelatinization RatePa·s /°C	Gel Viscosity (at 90 °C) Pa·s
P1	62.90 ± 0.50	0.515 ± 0.001	2.2 ± 0.5
P2	61.98 ± 0.09	2.3 ± 0.2	6.1 ± 0.9
P3	60.68 ± 0.40	5.3 ± 0.5	16.2 ± 1.5
P4	60.68 ± 0.65	16.2 ± 0.8	62.5 ± 2.0
P5	60.65 ± 0.58	22.8 ± 2.0	115.8 ± 7.2
P6	60.04 ± 0.40	32.4 ± 2.2	140.1 ± 5.2

**Table 2:** Color parameters, thickness, elongational at break, and elastic modulus for film samples.

Sample ID	L* −	a* −	b* −	Thickness mm	EAB% −	E MPa
P5	89.7 ± 0.1	1.7 ± 0.1	-2.5 ± 0.1	0.13 ± 0.02	37 ± 2	5 ± 1
W	55.8 ± 0.4	6.4 ± 0.3	28.6 ± 1.0	0.15 ± 0.02	7 ± 1	44 ± 1
DW	48.4 ± 0.4	5.1 ± 0.1	25.2 ± 1.0	0.17 ± 0.03	8 ± 1	53 ± 4
W_P	52.1 ± 0.4	5.3 ± 0.1	25.0 ± 0.1	0.24 ± 0.01	Not detectable	Too high
DW_P	50.8 ± 0.5	5.7 ± 0.4	28.6 ± 0.4	0.30 ± 0.03	17 ± 1	36 ± 1

**Table 3:** Contact angle value for tested film.

Sample ID	Contact Angle on Casting Surface, °	Contact Angle on Evaporation Surface, °
P5	17.0 ± 0.4	19.1 ± 0.5
W	44.5 ± 0.5	47 ± 3
DW	44 ± 1	61 ± 4
W_P	48 ± 4	54 ± 2
DW_P	50 ± 4	75 ± 2

**Table 4:** Composition of investigated samples.

ID	Extract % *w*/*w*	Starch % *w*/*w*	Glycerol % *w*/*w*	Water % *w*/*w*	Waste Water % *w*/*w*	Starch Added g/g_sospension_
P1	-	1	0.5	98.5	0	-
P2	-	2	1	97	0	-
P3	-	3	1.5	95.5	0	-
P4	-	4	2	94	0	-
P5	-	5	2.5	92.5	0	-
P6	-	6	3	91	0	-
W	4.8	-	2.4	0	94.8	-
DW	5	-	2.5	92.5	0	-
W_P	4.8	-	2.4	0	94.8	1.1/158
DW_P	4.8	-	2.4	92.5	0	2.2/98

## Data Availability

The data used to support the findings of this study can be made available by the corresponding author upon request.
